# Covalent drug rescue of multiple p53 mutants by stabilizing vinyl sulfone fragments hints to a specific refolding mechanism for R282W

**DOI:** 10.1002/pro.70629

**Published:** 2026-05-15

**Authors:** Jason Stahlecker, Theresa Klett, Robert Spiegel, Martin Schwer, Finn Mier, Sven Aldea, Benedikt Masberg, Michael Lämmerhofer, Thilo Stehle, Frank M. Böckler

**Affiliations:** ^1^ Lab for Molecular Design & Pharm. Biophysics, Institute of Pharmaceutical Sciences, Department of Pharmacy and Biochemistry Eberhard Karls Universität Tübingen Tübingen Germany; ^2^ Institute of Pharmaceutical Sciences, Pharmaceutical (Bio‐)Analysis University of Tübingen Tübingen Germany; ^3^ Interfaculty Institute of Biochemistry Eberhard Karls Universität Tübingen| Tübingen Germany

**Keywords:** cancer, cell cycle, covalent modification, stabilization, vinyl‐sulfone

## Abstract

The tumor suppressor protein p53 plays a crucial role in cell cycle regulation. In approximately 50% of all tumors, mutations in p53 are observed. Although the top hot spot mutations impair DNA binding, other mutations affect the thermal stability of the protein, leading to its rapid denaturation in physiological conditions. Previously, we have identified VS004, a vinyl sulfone containing fragment that stabilized p53C‐WT by about 5°C. In this study, we screened the CovLib fragments VS002, VS003, VS004, and two VS004 analogs against wild‐type p53C, as well as the mutants R273H, Y220C, and R282W. VS004 and its analogs demonstrated stabilizing effects on all tested p53 variants, with the strongest effect (almost 6°C) on R282W. We solved the crystal structures of VS004‐2 bound to Y220C and R282W. In the Y220C crystal structure, VS004‐2 bound to C277 and stabilized the loop L1. For R282W, we observed binding to C275, which leads to an unexpected structural reorganization of the C‐terminal helix H2. Although the arylated cysteines are both near the DNA‐binding interface, fluorescence polarization experiments using five different response elements confirm that DNA binding appears not to be compromised. The compounds present an interesting starting point for both general and mutant specific p53 stabilization.

## 1 INTRODUCTION

The tumor suppressor p53 plays an essential role in cell cycle regulation, apoptosis, senescence, and DNA‐repair, and for this, it is often referred to as the “guardian of the genome” (Joerger & Fersht, 2008; Lane, 1992; Levine et al., 2006; Vogelstein et al., 2000). In about 50% of all cancers, a mutation within p53 is observed (Kandoth et al., 2013; Leroy et al., 2014). Most of these mutations are located in the DNA‐binding domain (often referred to as Core domain) and are categorized as contact or structural mutants. The contact mutation class contains amino acids that are involved in direct DNA contact and binding, while the structural mutations do not directly interact with DNA but cause structural changes (Joerger & Fersht, 2008). The two most mutated residues are R248 and R273, both belonging to the group of contact mutants. Other commonly mutated residues are R175 (zinc binding region), G245, R282, R249 (all three DNA‐binding region), and Y220 (β‐sandwich) (De Andrade et al., 2022).

The Y220C mutation is the most common Y220 mutation and has been the focus of many previous studies (Baud et al., 2018; Bauer et al., 2016, 2019, 2020; Boeckler et al., 2008; Guiley & Shokat, 2023; Klett et al., 2025; Klett, Stahlecker, et al., 2024; Liu et al., 2013; Puzio‐Kuter et al., 2025; Stahlecker et al., 2022; Stephenson Clarke et al., 2022; Wilcken et al., 2012). This mutation causes a thermal destabilization of about 8°C (Bauer et al., 2020). Several generations of small molecules (PhiKan083 [Boeckler et al., 2008], PhiKan9318 [Bauer et al., 2019, 2020], PhiKan784 [Wilcken et al., 2012], PhiKan5196 [Wilcken et al., 2012], PhiKan7088 [Liu et al., 2013], MB725 [Baud et al., 2018] and many more) have been identified to rescue the protein by binding into the pocket and displacing the coordinated water molecules. With Rezatapopt, PMV pharma has currently a compound in clinical trials, which binds the cavity non‐covalently (Puzio‐Kuter et al., 2025). More recently, Guiley et al. have identified a covalently binding acrylamide that thermally almost fully rescues p53‐Y220C (Guiley & Shokat, 2023). We have also identified multiple fragment‐like small molecules (SN001, SN006, SN006/7‐3, SN006/7‐8, SN006/7‐9 [Klett, Stahlecker, et al., 2024; Klett et al., 2025]) containing S_N_Ar warheads that stabilize the Y220C mutant by typically mimicking the native tyrosine (Klett et al., 2025; Klett, Stahlecker, et al., 2024).

Unlike Y220C, the R282W mutant has not been in the focus of drug discovery trials. The arginine, located in the C‐terminal helix H2 in the DNA‐binding region, stabilizes the loop‐sheet‐helix region. Upon mutation to the bulkier tryptophan, the protein is destabilized by about 3 kcal/mol, leading to rapid denaturation under physiological conditions (Bullock et al., 2000). The mutation is considered a gain of function mutation and significantly impacts patient survival (Xu et al., 2014; Zhang et al., 2016).

While most research has focused on finding fragments that bind to the Y220C specific crevice for stabilization, some compounds capable of pan‐mutant p53 rescue have been found. The small molecule PRIMA‐1 has been identified to rescue multiple different mutants (Bykov et al., 2002). Later, the crystal structures with and without DNA across multiple p53 mutants have shown that PRIMA‐1 alkylates the cysteines C124, C182, C229, C273, C275, and C277, acting as a prodrug that is metabolized into the biologically active form methylene quinuclidinone (Degtjarik et al., 2021). The class of 2‐sulfonylpyrimidines has also been shown to arylate and stabilize p53, independently of its mutational status (Bauer et al., 2016; Pichon et al., 2023). The arylation sites of C182 and C277 have both been identified in the crystal structures.

We have previously presented a covalent fragment library (CovLib) and have discussed multiple hits and SAR on Y220C (Klett et al., 2025; Klett, Schwer, et al., 2024; Klett, Stahlecker, et al., 2024). During the initial screen, we observed the promising hit VS004, stabilizing p53C‐WT by about 5°C. These results intrigued us to test whether the warhead class of vinyl sulfones and especially VS004 can be starting points for pan‐mutant stabilizers. For this, we have chosen to screen the two thermally destabilized mutants Y220C and R282W, as well as the DNA‐contact mutant R273H (and WT) to better understand possible mechanisms of thermal rescue. The compound VS001 was omitted from further investigation, as the purity remained unclear for reasons discussed in Klett et al. (Klett, Schwer, et al., 2024).

## 2 RESULTS AND DISCUSSION

**FIGURE 1:**
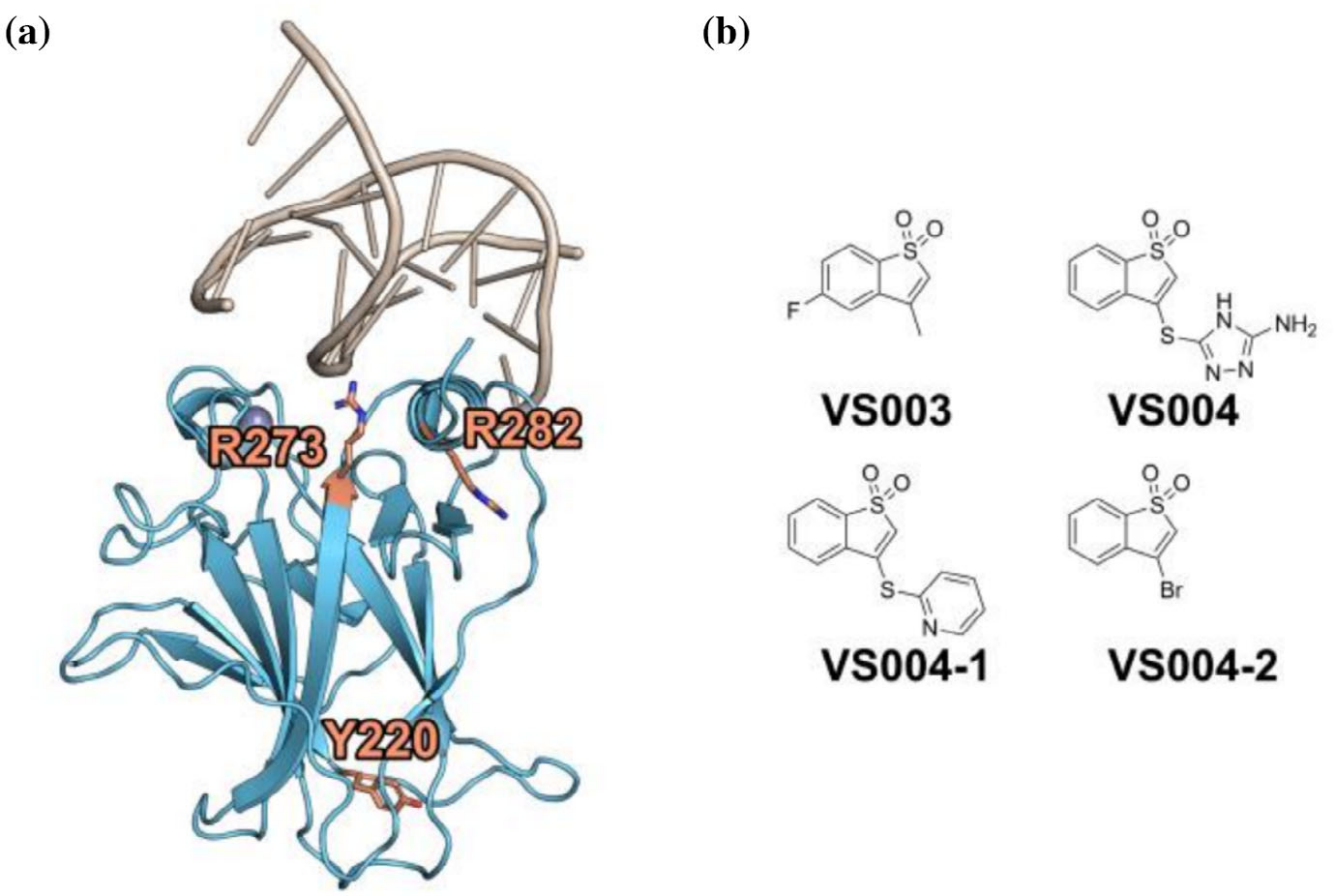
General Structure of the p53 Core Domain bound to DNA as well as a structural representation of the investigated fragments. (a) Binding of the core domain of p53 to DNA. The mutated residues discussed in this paper are highlighted, namely R273H, R282W and Y220C. (b) Structures of the four investigated fragments.

We investigated the thermal stabilization properties of VS002, VS003, and VS004 with a differential scanning fluorimetry (DSF) based screen against the tetra stabilized core domain of p53 (T‐p53C) (Nikolova et al., 1998) with the additional cancer mutations being either Y220C, R282W, or R273H (Figure 1).

As in the previous screens, compound VS002 did not result in interpretable DSF curves and was therefore omitted from our investigations. The results of the thermal stabilization are summarized in Table 1.

**TABLE 1:** Summary of the melting temperatures Δ*T*_*m*_ after 30 min of incubation. All numbers are in units of K.

Compound	WT	Y220C	R282W	R273H	Y220C‐CL
VS003	−0.05 ± 0.09^a^	−0.10 ± 0.21	−0.10 ± 0.12	−0.05 ± 0.17	N/D^b^
VS004	5.06 ± 0.09^a^	3.00 ± 0.16	5.96 ± 0.10	5.37 ± 0.12	−0.30 ± 0.19
VS004‐01	5.40 ± 0.26	3.33 ± 0.11	6.55 ± 0.09	5.80 ± 0.09	0.40 ± 0.27
VS004‐02	4.55 ± 0.38	3.33 ± 0.15	5.75 ± 0.17	4.85 ± 0.35	0.95 ± 0.12

^a^ Data obtained from (Klett, Schwer, et al., 2024).

^b^ The melting temperature of Y220C‐CL was not determined with VS003.

While VS003 did not thermally stabilize any mutant, VS004 stabilized all mutants. The low stabilization property of VS003 can be explained by its low reactivity as assessed by a glutathione (GSH) assay (Klett, Schwer, et al., 2024). The highest thermal stabilization was observed with the R282W mutant by up to almost 6°C, resulting in a near full thermal rescue (Table S1). As the Δ*T*_*m*_ for all tested mutants, except Y220C, was in a similar range, and even wild type (WT) was strongly stabilized, we expected a common, mutant‐independent mechanism of stabilization.

We had previously observed addition and elimination products in the intact protein mass spectrometry (MS) spectra of VS004 binding to T‐p53C‐WT suggesting modifying effects of groups attached to the electrophilic carbon atom in position 3 of the benzothiophene sulfone scaffold (benzo[*b*]thiophene 1,1‐dioxide). Taking this and the propensity of pan‐mutant stabilization into consideration, we tested compounds VS004‐1 and VS004‐2 (Figure 1) to characterize the influence of altered groups on the extent of stabilization. Both SAR compounds stabilize the proteins, including the WT, to a similar extent as VS004 (Table 1) with compound VS004‐1 having a slightly stronger effect than VS004 or VS004‐2. In a GSH reactivity assay, we obtained t_1/2_ rates of 0.62 ± 0.004 h for VS004‐1 and 0.5 ± 0.01 h for VS004‐2, respectively. Results and graphs for GSH as well as PBS reactivity are presented in Figure S1 and Table S2. Both VS004‐1 and VS004‐2 are only slightly more reactive than VS004 (0.82 ± 0.03 h).

To confirm that the compounds VS004‐1 and VS004‐2, like VS004, covalently bind to T‐p53C‐WT we performed intact protein MS and the resulting spectra are presented in Figure S2. Although we observed unmodified protein for p53C‐WT preincubated with VS004‐1, the spectrum suggests that the compound is highly reactive and multiple elimination and addition species were observed. For VS004‐2 we only observed two‐ and three‐fold elimination products. Both compounds show high reactivity with GSH and multiple arylations were expected. As bromine is a good leaving group, addition products were not expected for VS004‐2. This is in line with the experimental data. It is likely that the attached (leaving‐) group has a strong impact on the arylation pattern and the GSH reactivity can only estimate general reactivity.

We then performed X‐ray crystallography to elucidate the binding mode and identify the origin of the stabilization. We decided to soak VS004‐2, as it presents a good balance between size, solubility, reactivity, and thermal stabilization and obtained a dataset for the mutants Y220C (pdb_00009szz) and R282W (pdb_00009suk). The final statistics are presented in Tables S3 and S4.

**FIGURE 2:**
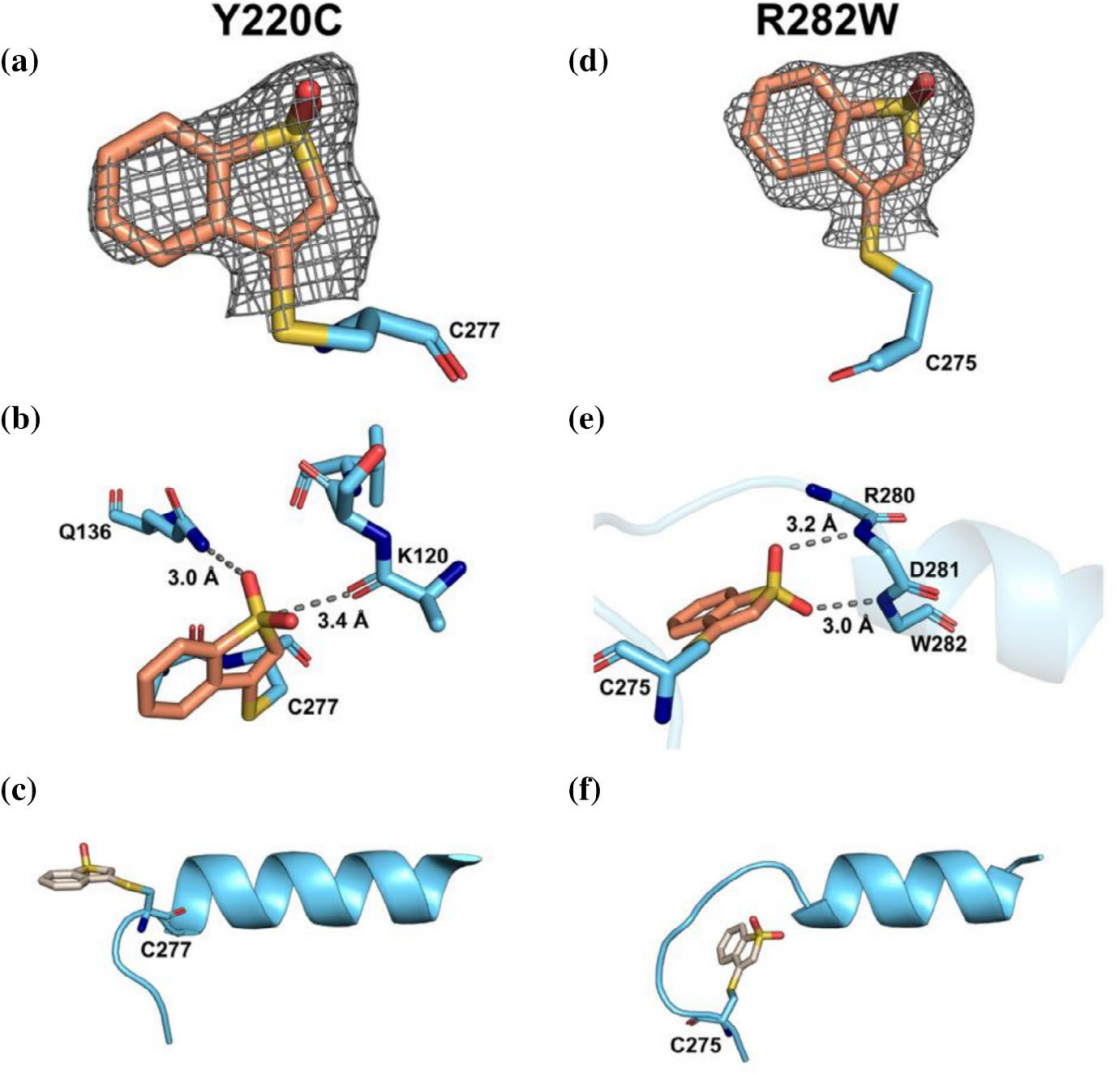
The unbiased omit map, contoured at 3 σ is presented for the Y220C mutant in (a) and for the R282W mutant (pdb_00009SUK) in (d). (b) For mutant Y220C the ligand interacts with Q136 via a hydrogen bond, as well as the backbone K120 of loop L1. The S—O interaction distance is marked to show the close distance. (e) In the R282W mutant however, the ligand covalently binds C275 rather than C277 and interacts with the backbone nitrogen of D281 and W282 in the C‐terminal helix. (c) and (f) compare the structural rearrangement observed in the R282W mutant by unwinding the helix and stabilizing it by interactions shown in (e).

In the structure of the Y220C mutant, we observed covalent modification of C277 (Figure 2a). The compound interacts with the nitrogen of the carboxamide group of Q136 via a hydrogen bond (Figure 2b). It also interacts with the backbone oxygen of K120 of loop L1 with an S—O distance of 3.4 Å (Figure 2b). The residue C277 is located at the beginning of the helix H2 and has often been observed to be covalently modified (Degtjarik et al., 2021; Klett et al., 2025; Klett, Stahlecker, et al., 2024; Pichon et al., 2023). Unlike the MS data, we only observed a single modification in the crystal structure. No further unmodeled density can be explained by another binding of the compound VS004‐2. Binding to other cysteines could be hindered due to altered kinetics or contacts within the crystal. The observed binding mode is not influenced by crystal contacts. The interaction with loop L1 can be a reason for the mutant independent stabilizing effect.

For the investigation of the close contact of two electro‐negative moieties (sulfonyl group of the ligand and carbonyl of K120, Figure 2b) we calculated an ESP of the ligand, showing a σ‐hole of the sulfonyl group oriented toward the carbonyl to form a chalcogen bond (Figure S3). Decomposition of the interaction energy using a CovaLED analysis indicates that the sulfonyl group's interaction with the carbonyl is indeed a main contributor (Figure S3 and Table S5).

The mutant R282W has a destabilized loop‐sheet‐helix region, and we expected that binding to C277 would ultimately stabilize this region, and we would observe density for the strongly destabilized loop (in R282W). In fact, we did observe some partial density around C277 in chain A that the compound can explain and modeled it with partial occupancy. However, in chain B, we observed a different, unexpected binding mode.

The effect of the binding of the compound is rather remarkable as it rearranges the C‐terminal helix H2. The compound binds to C275 (Figure 2d) and interacts with the backbone amines of the helix. The interaction distances and angles of the sulfonyl group seem to mimic the backbone carbonyls of the typical H‐bond interactions within an ⍺‐helix (Figure 2e). When comparing the helix in bound form of R282W (Figure 2f) to Y220C (Figure 2c), the first turn is unwound and loops over the compound.

We suspect that this mode of stabilization is unique to this mutant as the missing arginine destabilizes this region and enables rearrangement of the helix. Again, this region does not have any close crystal contacts that could be a driving force or cause of this rearrangement. We note that the average ligand B‐factor (66.1 Å^2^) is well above the average protein B‐factor (30.8 Å^2^). The value of the ligand bound to C275 in chain B is 62 Å^2^, while the average B‐factor of the protein within 3.5 Å is 61 Å^2^. This suggests that the entire region is more flexible than other parts of the protein, which is in line with the observation of Joerger et al. (Joerger et al., 2006). Additionally, the ligand in chain A, bound to C277, was entirely built although complete density was not observed, because the positions of the benzene ring are chemically fully defined by the ligand geometry. This combination increases the B‐factor but does not disprove or challenge the binding and rearrangement of the final model.

Too narrow down the origin of the stabilization, we performed DSF measurements with p53C‐Y220C‐CL (C124/182/229/275/277S), a mutant lacking all solvent accessible cysteines. The results are presented in Table 1. Under the same conditions as p53C‐Y220C, p53C‐Y220C‐CL is only stabilized by VS004‐2, by up to about 1°C. We acknowledge that thermal stabilization may be a complex interplay of different arylation events and thus altered interactions and kinetics. Nonetheless, it highlights the possible effects of C275 and C277.

**FIGURE 3:**
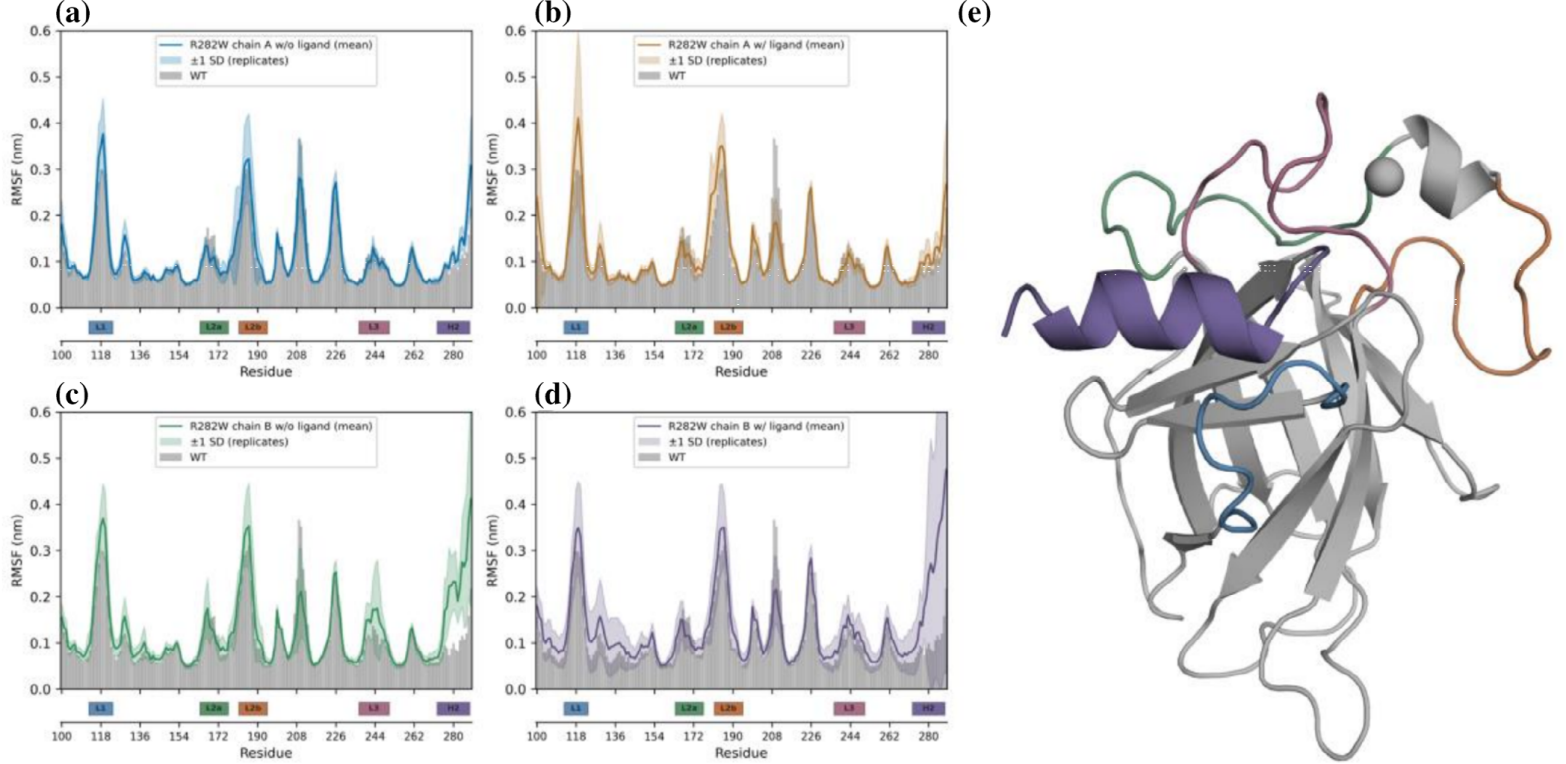
Residual flexibility profile of chain A and B (9SUK), with and without receptor compared to WT, as indicated by root mean square fluctuation (RMSF). The mean RMSF between replicas is shown as a solid line and the 1 standard deviation between replicas is indicated with a shaded band. In the background of each plot, the RMSF of the WT is indicated by gray bars. (a) Chain A, but the ligand was removed prior to simulation setup. (b) Chain A with ligand as in the crystal structure. (c) Chain B, but the ligand was removed prior to simulation setup. (d) Chain B with ligand as in the crystal structure. (e) Color coding of the loops and helix H2 as used in (a)–(d).

We wanted to get an understanding of the proteins dynamics and ran short molecular dynamics (MD) simulations (8 replicas of 200 ns per structure) of chain A and chain B of structure 9SUK (p53‐R282W bound to VS004‐2) with and without ligand. These were compared to a simulation of a wild‐type protein (2OCJ) (Wang et al., 2007). Simulations yielded results consistent with the literature, suggesting general stability of the protein (Calhoun & Daggett, 2011; Lukman et al., 2013). Root‐mean‐square fluctuation (RMSF) analysis indicates increased local flexibility for the mutant structures in the H2 helix adjacent to the mutation site (Figure 3). This effect is particularly pronounced in the chain B ligand‐bound structure, in which ligand binding is associated with a partially unwound H2 helix. The apparently elevated flexibility in the structure of chain B upon removing the ligand likely reflects the initially unwound starting conformation, which can refold during the simulation, yielding fully restored helices in some replicas. Additional plots of the MD simulation are presented in the supplementary information Figures S4–S8.

**FIGURE 4:**
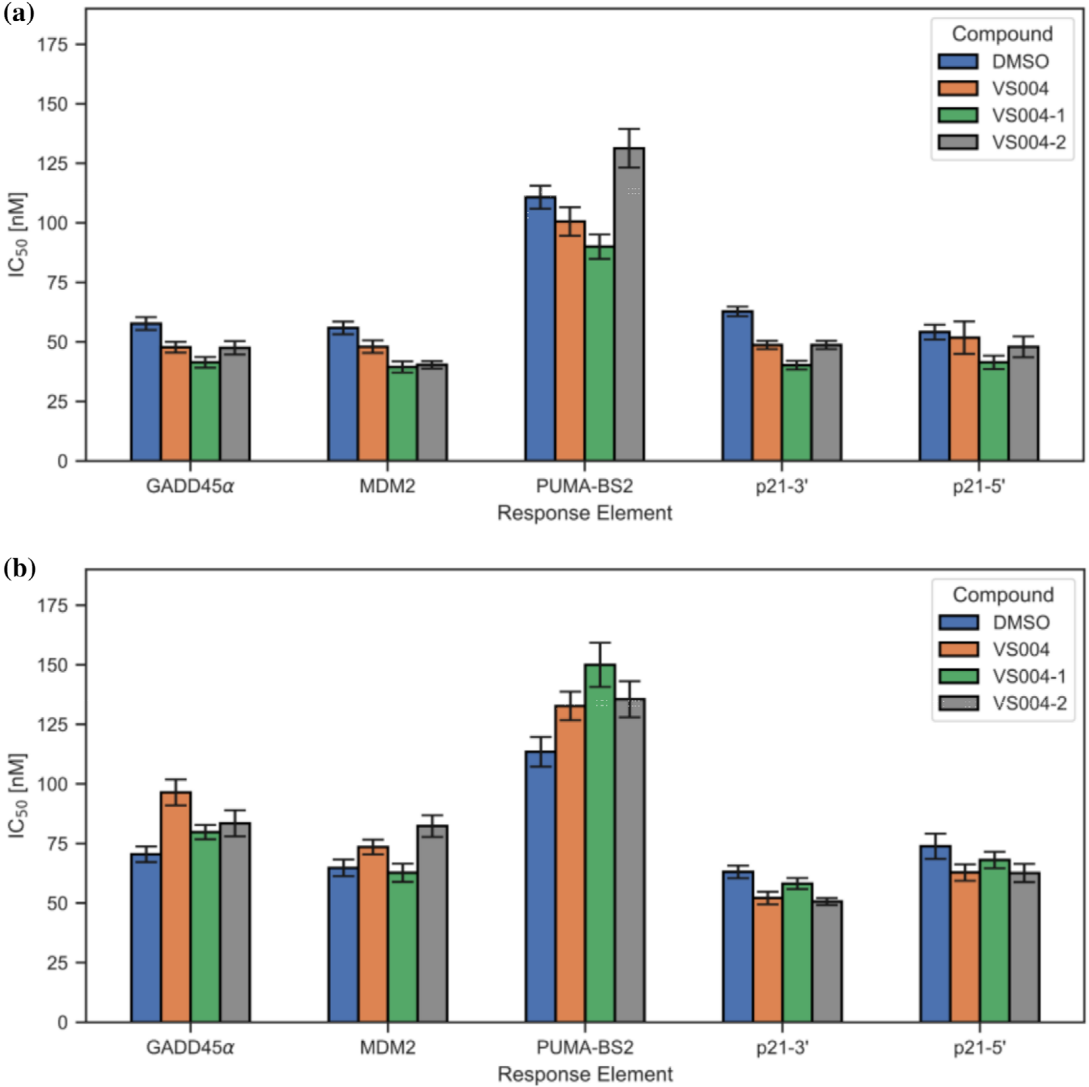
K_D_ values for the binding of response elements (GADD45α, MDM2, PUMA‐BS2, p21‐3′, and p21‐5′) to (a) FL‐Tp53‐WT and (b) FL‐Tp53‐R282W, following 1 h. incubation with DMSO, VS004, VS004‐1, or VS004‐2.

Next, we were interested whether binding to either C277 or C275 would affect binding of tetra stabilized full length p53 (FL‐T‐p53) to DNA as both cysteines are within or close to the DNA‐binding interface. We performed a fluorescence polarization assay with the fluorescein‐tagged response elements GADD45α, MDM2, PUMA‐BS2, p21‐3′, and p21‐5′ site (Weinberg et al., 2005) to FL‐T‐p53‐WT and FL‐T‐p53‐R282W, both preincubated with VS004, VS004‐1, and VS004‐2 for 1 h. The final K_D_ values are displayed in Figure 4 and summarized in Table S6. The individual fits are displayed in Figures S9 and S10.

While binding to all response elements is maintained, some minor alterations of affinity are observed when compared to DMSO. Thus, in principle, mutant rescue of FL‐T‐p53‐R282W could lead to activation of the corresponding downstream effects. For the response elements GADD45α and p21‐5′, we additionally performed measurements after 24 h of preincubation time between the rescuing ligands and the R282W mutant. While the overall affinity of the R282W mutant (stabilized or not) is slightly worse after 24 h, only rather irrelevant changes are observed between the unstabilized (DMSO) and the stabilized (VS004, VS004‐1, and VS004‐2) R282W mutant (Figure S11 and Table S7). This seems promising as it appears that in vitro, a modification of C277 and/or C275 does not disrupt the DNA binding, essential for p53's regulatory functions.

## 3 CONCLUSION

The vinyl sulfone warhead‐containing compound VS004 showed promising effects in our initial screen. The compounds VS004‐1 and VS004‐2 showed very similar stabilization profiles as the binding mode will likely be the same, but differences in kinetics as well as the equilibrium of addition versus substitution will have an effect. The crystal structures of VS004‐2 bound to mutant Y220C and R282W shed light on two different stabilizing mechanisms.

We speculate that the binding mode observed in the Y220C mutant could depict the more generalized stabilization mechanism. The fragment interacts with the L1 loop in an atypical sulfonyl–carbonyl contact as well as a typical hydrogen bond with a neighboring glutamine. It is likely that this dual interaction rigidizes the region leading to protein stabilization.

In mutant R282W, where the loop‐sheet‐helix region is uniquely destabilized, fragment binding causes a structural rearrangement of this C‐terminal helix H2, which may be the origin of the thermal rescue leading to a thermal rescue.

Such sort of helical rearrangement, especially with a mimic of the natural backbone, has, to the best of our knowledge, not been observed before. It might be speculative, yet potentially rewarding, to assess in the future whether such an effect could be utilized more systematically in peptide/protein design.

Although both arylations could affect DNA binding, data show no compromising effect. In general, the warhead class of vinyl sulfones shows promising starting points for general as well as R282W‐specific stabilization. As these chemical probe‐like fragments are highly reactive, we do not expect promising results in cellular setups. Another consequence of the high reactivity is the multiple addition as seen in the MS experiments, which will need to be overcome.

We also note that the current compounds are too reactive to expect promising results in cellular setups. The modulation of reactivity as well as creating more specific interactions will be key for further improvement.

We cannot exclude that for WT, arylation of R275 or for R282W the arylation of R277 may also contribute to the observed thermal rescue. Elaborate in solution structure elucidation could shed light into this.

## 4 MATERIALS AND METHODS

All Chemical were purchased from standard vendors.

### 4.1 Expression and purification

All core domain mutants were expressed and purified as previously published (Stahlecker et al., 2022). The full length WT was expressed and purified as described elsewhere (Klett, Stahlecker, et al., 2024). For the R282W mutant, we encountered high DNA absorption and adapted the protocol. The lysis buffer was supplemented with 1 M of NaCl. The lysate was incubated with DNAse and RNAse for 4 h at 4°C before loading onto the Ni‐NTA column.

### 4.2 X‐ray crystallography

For crystallography, a shortened construct for the core domain mutants was used (Stahlecker et al., 2022). Sitting drop vapor diffusion with streak seeding was performed to obtain crystals for both the Y220C and R282W mutants. The protein was concentrated to 5 mg/mL and mixed in a 1:1 ratio with the reservoir solution (100 mM HEPES [pH = 7.2], 19% PEG4000, and 10 mM DTT). For soaking, crystals were transferred to a cryoprotectant solution containing 20% glycerol and 5 mM compound. Soaking was performed overnight and then flash frozen in liquid nitrogen for storage.

Datasets were obtained at the Swiss Light Source (SLS) at the beamline X06SA (PXIII). Data reduction and scaling were performed using XDS (Kabsch, 2010). Initial phases were obtained by molecular replacement using PHASER as part of the ccp4 suite (McCoy et al., 2007; Winn et al., 2011). For the Y220C mutant 8A92 (Stahlecker et al., 2022) was used as the search model, while for the R282W mutant 2 J21 (Joerger et al., 2006) was used. For model and phase improvement, multiple rounds of manual model building in coot and structure refinement in PHENIX were performed (Emsley et al., 2010; Liebschner et al., 2019). Ligand restraints were generated using JLigand in ACEdrg mode (Lebedev et al., 2012; Long et al., 2017).

### 4.3 Differential scanning fluorimetry

All DSF was conducted on a Qiagen Rotor‐Q Model 5plex HRM real‐time PCR instrument as previously described (Stahlecker et al., 2022). The protein (8 μM) was incubated with compound (1 mM) or DMSO for 30 mins (5% final DMSO conc. (v/v)). The triplicate and averaged *T*_*m*_ values were obtained by calculating the maximum of the first derivative using OriginPro2020 (OriginLab, Northampton, MA, USA). To obtain Δ*T*_*m*_ values, the reference value (incubation with DMSO) was subtracted from the compound incubated samples using error propagation theory. For measurements to obtain mutant specific Δ*T*_*m*_, we performed sextuplicate measurements of T‐p53C, T‐p53C‐R282W, T‐p53C‐Y220C, and T‐p53C‐R273H.

### 4.4 Fluorescence polarization assay

The FP‐assays were performed as described elsewhere applying a 1 or 24 h preincubation time (protein‐to‐compound ratio of 1:200) (Klett, Stahlecker, et al., 2024). Instead of 96‐well plates, 384‐well plates (GBO, Frickenhausen, Germany) were used. Triplicates were averaged over time. Background signal was subtracted using the no‐protein control, with error propagation handled using the uncertainties Python package (Leibigot, n.d.). Normalized data were subsequently fitted to a Hill equation including a linear drift term, without weighting individual data points by their measurement error. Curve fitting was performed using the SciPy Python package (Bauer et al., 2016; Kaar et al., 2010; Weinberg et al., 2004).

### 4.5 Intact protein MS

All intact protein MS measurements and evaluation were performed as in (Klett, Stahlecker, et al., 2024). The protein T‐p53C‐WT was preincubated with VS004‐1 and VS004‐2 with a molar ratio of 1:100 (5% DMSO) for 24 h. The spectra were recorded using a UHPLC‐ESI‐MS system with a time‐of‐flight analyzer (TripleTOF 5600+, Sciex) using an intact protein MS script.

### 4.6 GSH assay

All GSH reactivity studies were performed as previously described (Klett, Schwer, et al., 2024). In brief, the reaction conditions were PBS pH 7.4, 10% acetonitrile, 100 μM ketoprofen (internal standard), 250 μM fragment, and 5 mM GSH excess at 37°C. Measurements were taken after 0, 1, 2, 4, 8, 12, and 24 h and loaded onto an Ultimate 3000 HPLC‐System (Thermo Fisher Scientific, Dreieich, Germany). The resulting graphs were fitted using OriginPro2020 using a pseudo‐first order kinetics function.

### 4.7 QM calculations

To investigate the sulfonyl's interaction, we carried out a single point interaction energy calculation using ORCA 6.1.0 (Neese, 2025) on the DLPNO‐CCSD(T)/def2‐TZVPP level (Riplinger et al., 2013; Riplinger & Neese, 2013) between the ligand and subsets of the K120 (C, O, C_α_, C_β_, C_γ_) and S121 (N, Cα) residues, decomposing the interaction energy using the CovaLED scheme (Altun et al., 2025). The calculation used VeryTightSCF and TightPNO convergence settings. Additionally, we computed the ligands' electrostatic potential on the MP2/def2‐TZVPP level (Feyereisen et al., 1993; Møller & Plesset, 1934) with TightSCF convergence settings. The calculations were preceded by adding missing hydrogens and their geometric optimization on the B3LYP/SVP level (Becke, 1993; Stephens et al., 1994) with Grimme's D4 dispersion correction (Caldeweyher et al., 2020) and TightSCF convergence settings. All calculations used the resolution of identity and chain‐of‐spheres exchange algorithm (Izsák et al., 2012) approximations.

### 4.8 MD simulations

MD simulations were carried out for chain A and chain B of 9SUK with and without ligand, as well as a wild‐type protein structure (PDB: 2OCJ) (Wang et al., 2007). Each protein was considered as a monomer, keeping only the protein, ligand, and Zn^2+^ ion. All structures were prepared and protonated using Schrodingers Protein Preparation Wizard (Schrödinger Release 2026‐1) (Madhavi Sastry et al., 2013). The missing L1 residues (GTAKS for chain A and SGTAKSV for chain B) were added with Schrodingers Protein Linker Design using PRIME with implicit solvent (Jacobson et al., 2004). CHARMM‐GUI's Solution Builder was used to generate a TIP3P water box with an Edge distance of 10 Å, including Potassium and Chlorine ions at 0.15 mol/L concentration (Brooks et al., 2009; Jo et al., 2008; Jorgensen et al., 1983; Lee et al., 2016). The ligand was parameterized using CGenFF (Vanommeslaeghe et al., 2010).

For each structure 8 replicates were simulated using GROMACS with the CHARMM forcefield (Abraham et al., 2015, 2024; Brooks et al., 2009). After restrained steepest‐descent energy minimization (5000 steps), the systems were equilibrated for 125 ps in the NVT ensemble at 303.15 K with positional restraints on solute backbone (400.0 kJ mol^−1^ nm^−2^) and side‐chain atoms (40.0 kJ mol^−1^ nm^−2^). Production MD was then performed for 200 ns with a 2 fs time step in the NPT ensemble at 303.15 K and 1 bar, using the v‐rescale thermostat and C‐rescale barostat (Bernetti & Bussi, 2020; Bussi et al., 2007). Electrostatics were treated with PME, short‐range electrostatic and van der Waals cutoffs were set to 1.2 nm, and a force‐switch was applied to van der Waals interactions from 1.0 to 1.2 nm (Darden et al., 1993). Hydrogen‐containing bonds were constrained using LINCS.

## AUTHOR CONTRIBUTIONS

**Frank M. Böckler:** Conceptualization; project administration; resources; supervision; writing – original draft; funding acquisition. **Jason Stahlecker:** Data curation; investigation; validation; formal analysis; project administration; visualization; writing – original draft. **Benedikt Masberg:** Formal analysis; investigation; writing – review and editing; data curation. **Finn Mier:** Data curation; formal analysis; investigation; visualization; writing – review and editing. **Michael Lämmerhofer:** Writing – review and editing; resources; supervision. **Thilo Stehle:** Resources; writing – review and editing; supervision. **Robert Spiegel:** Data curation; formal analysis; investigation; writing – review and editing. **Sven Aldea:** Data curation; investigation; formal analysis; writing – review and editing. **Theresa Klett:** Data curation; investigation; formal analysis; project administration; writing – review and editing. **Martin Schwer:** Data curation; formal analysis; investigation; visualization; writing – review and editing.

## CONFLICT OF INTEREST STATEMENT

The authors declare no competing interests.

## Supporting information


**Data S1.** Supporting information.

## Data Availability

The data that support the findings of this study are available from the corresponding author upon reasonable request.
