# Supplementary material for: Covalent drug rescue of multiple p53 mutants by stabilizing vinyl sulfone fragments hints to a specific refolding mechanism for R282W
Source: Protein Sci. 2026 May 15;35(6):e70629. doi: 10.1002/pro.70629 (PMC13179279; doi:10.1002/pro.70629)
Supplement: Supplementary file 1 — Data S1. Supporting information. [file PRO-35-e70629-s001.pdf]

Covalent Drug Rescue of Multiple p53 Mutants by Stabilizing Vinyl Sulfone Fragments Hints to a Specific Refolding Mechanism for R282W

Jason Stahlecker[a], Theresa Klett[a], Robert Spiegel[a], Martin Schwer[a], Finn Mier[a], Sven Aldea[a] Benedikt Masberg[b], Michael Lämmerhofer[b], Thilo Stehle[c] and Frank M. Boeckler[a,d,*]

[a] Lab for Molecular Design & Pharm. Biophysics, Institute of Pharmaceutical Sciences, Department of Pharmacy and Biochemistry, Eberhard Karls Universität Tübingen, Auf der Morgenstelle 8, 72076 Tübingen, Germany

[b] Institute of Pharmaceutical Sciences, Pharmaceutical (Bio-)Analysis, University of Tübingen, Auf der Morgenstelle 8, Tübingen, Germany

[c] Interfaculty Institute of Biochemistry, Eberhard Karls Universität Tübingen, Auf der Morgenstelle 34, 72076 Tübingen, Germany

[d] Interfaculty Institute for Biomedical Informatics (IBMI), Eberhard Karls Universität Tübingen, Tübingen, Germany

*Corresponding Author: Frank M. Böckler. Auf der Morgenstelle 8, 72108

Tübingen. Tel: +49 7071 29 74567. frank.boeckler@uni-tuebingen.de

Email: frank.boeckler@uni-tuebingen.de

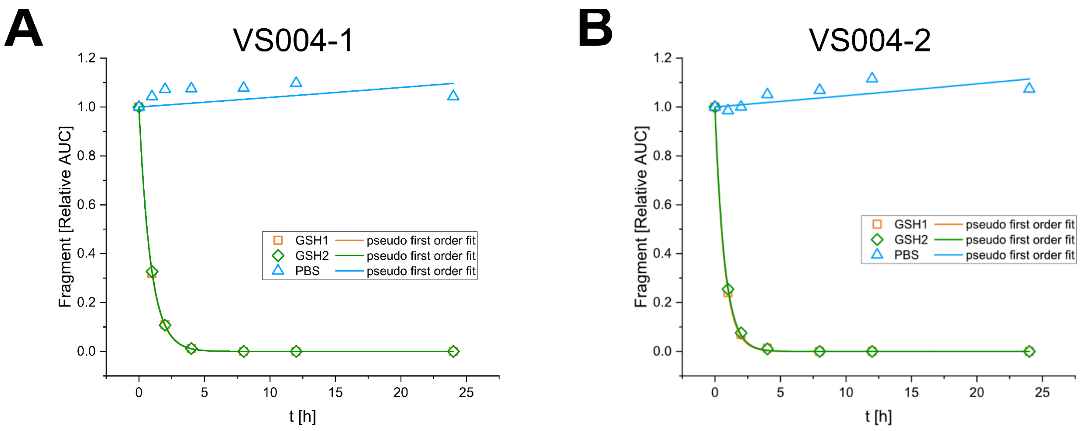

Fig. S1. Results of the reactivity measurements of A) VS004-1 and B) VS004-2 with GSH and PBS.

Table S1. Results of the DSF measurements comparing the T-p53C mutants R282W, Y220C, and R273H to T-p53C(-WT).

Mutant	ΔT_m [K]
R282W	– 6.75 ± 0.00[a]
Y220C	– 7.43 ± 0.08
R273H	– 0.85 ± 0.08

[a] When determining ΔT_m for the R282W mutant, all 12 curves (6x WT and 6x R282W) resulted in the same melting temperature.

Table S2. Data of the reactivity measurements of VS004-1 and VS004-2 with GSH and PBS

Compound	$t_{1/2}$ GSH [h]	$t_{1/2}$ PBS [h]
VS004-1	0.62 ± 0.004	– 181[a]
VS004-2	0.50 ± 0.01	– 153[a]

[a] Negative values arise from curve fitting (see Figure S1) and indicate that the compound is stable over time.

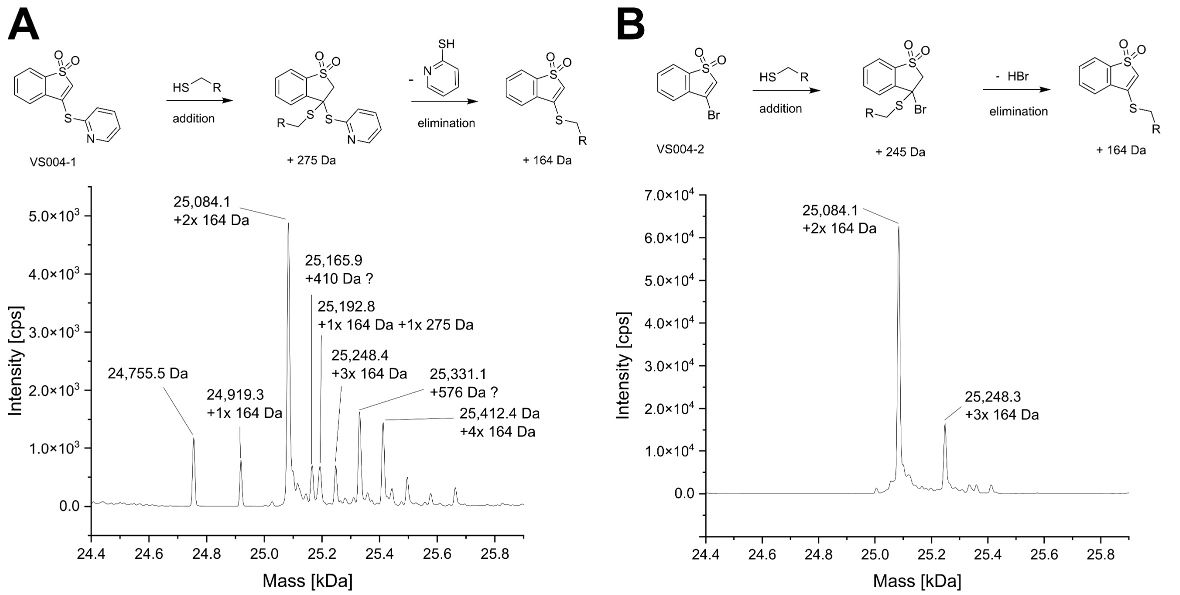

Fig. S2. MS results of T-p53C-WT incubated with A) VS004-1 and B) VS004-2. VS004-1 had multiple peaks with some not able to be assigned and unmodified protein was observed. The spectrum of VS004-2 shows double and triple arylated protein.

Table S3. Data collection and refinement statistics of Tp53C-Y220C soaked with 5 mM VS004-2 (PDB: 9SZZ, pdb_00009szz)

Wavelength [Å]	1
Space Group	19
Cell Dimensions	
a / b / c [Å]	65.10 / 71.05 / 105.27
alpha / beta / gamma [°]	90.00 / 90.00 / 90.00
Resolution Range [Å]	50.00 - 1.70 (1.80 - 1.70)
Redundancy	12.51 (8.17)
Completeness [%]	99.80 (98.70)
Mean I/sigma(I)	12.17 (1.01)
R_{meas}	12.30 (188.10)
CC ½ [%]	99.90 (65.50)
Wilson B factor [Å^2]	34.29
Resolution Included [Å]	48.00 - 1.70
R_{work}/R_{free}	17.56 / 21.28
Bond RMSD [Å]	0.01
Angle RMSD [°]	1.003
Ramachandran Favored / Allowed / Outliers	99.75 / 0.25 / 0.00
Rotamer Outliers [%]	2.06
All Atom Clashscore	1.62
Average B factor [Å^2]	
Overall	33.7
Protein	32.2
Water	41
Ligand	49.1

Table S4. Data collection and refinement statistics of Tp53C-R282W soaked with 5 mM VS004-2 (PDB: 9SUK, pdb_00009SUK)

Wavelength [Å]	1
Space Group	19
Cell Dimensions	
a / b / c [Å]	65.47 / 71.18 / 105.01
alpha / beta / gamma [°]	90.00 / 90.00 / 90.00
Resolution Range [Å]	50.00 - 1.56 (1.65 - 1.56)
Redundancy	11.17 (10.96)
Completeness [%]	100.00 (100.00)
Mean I/sigma(I)	13.52 (1.00)
R_{meas}	13.00 (264.30)
CC ½ [%]	99.90 (63.80)
Wilson B factor [$Å^2$]	30.54
Resolution Included [Å]	48.19 - 1.56
R_{work}/R_{free}	15.87 / 20.62
Bond RMSD [Å]	0.009
Angle RMSD [°]	0.971
Ramachandran Favored / Allowed / Outliers	99.20 / 0.80 / 0.00
Rotamer Outliers [%]	2.54
All Atom Clashscore	2.52
Average B factor [$Å^2$]	
Overall	33.3
Protein	30.8
Water	44.0
Ligand	66.1

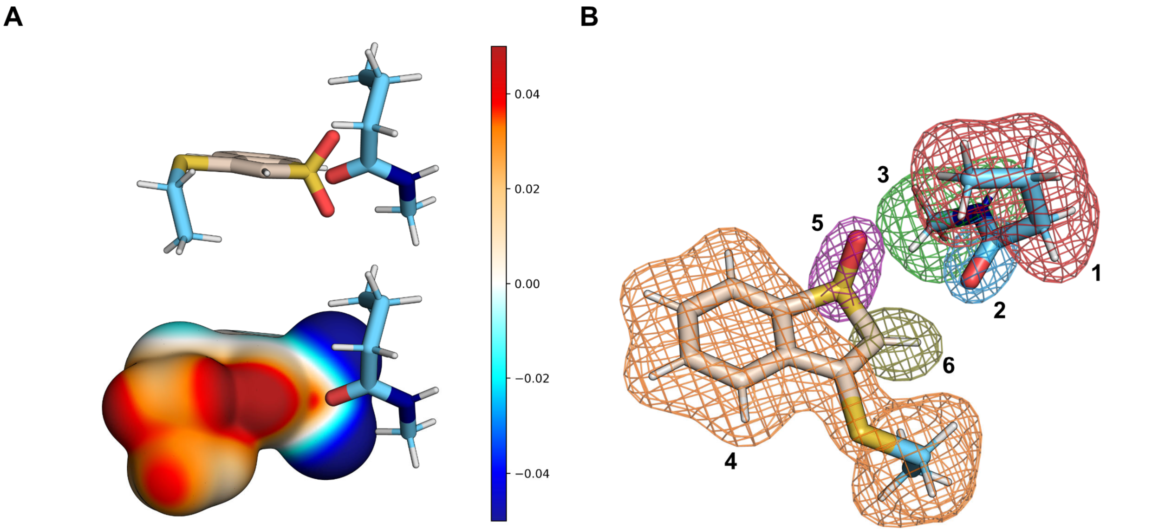

Fig. S3. Visual representations of the ESP calculation and CovaLED analysis. A) The ESP is plotted on the isosurface at 0.002 au. The colorbar is in units of au (E_h/e). B) The fragment decomposition and their numbering used for table S5 is displayed.

Table S5. Results of the CovaLED decomposition. All units are in kJ/mole. The strongest two interactions between the Bound ligand (Fragments 4+5+6) and the Backbone of Loop L1 (Fragments 1+2+3) are marked in bold.

	Red (1)	Blue (2)	Light Green (3)	Orange (4)	Purple (5)	Dark Green (6)
Red (1)	16.18	-4.80	3.41	-1.90	-21.69	0.55
Blue (2)		16.83	-54.27	-21.37	**-30.32**	-5.40
Light Green (3)			72.66	-12.26	**-33.63**	-0.32
Orange (4)				-14.80	15.41	-95.97
Pruple (5)					11.13	-22.26
Dark Green (6)						149.64

Table S6. IC_{50} values of the response elements binding to WT or R282W prior to compound or DMSO incubation (1 hour). All values are presented in units of nM.

	WT				R282W			
	DMSO	VS004	VS004-1	VS004-2	DMSO	VS004	VS004-1	VS004-2
PUMA	111 ± 5	101 ± 6	90 ± 5	131 ± 8	113 ± 6	133 ± 6	150 ± 9	136 ± 8
MDM2	56 ± 3	48 ± 3	39 ± 2	40 ± 2	65 ± 3	73 ± 3	63 ± 4	82 ± 5
GADD45α	58 ± 3	48 ± 2	41 ± 2	47 ± 3	70 ± 3	96 ± 5	80 ± 3	83 ± 5
P21-3’	63 ± 2	49 ± 2	40 ± 2	49 ± 2	63 ± 3	52 ± 3	58 ± 2	51 ± 1
P21-5’	54 ± 3	52 ± 7	41 ± 3	48 ± 4	74 ± 5	63 ± 3	68 ± 3	63 ± 4

Additional MD Analyses:

In addition to RMSF analyses we also tracked solvent accessible surface of the core protein and the core RMSD of the protein. No major differences to WT were observed.

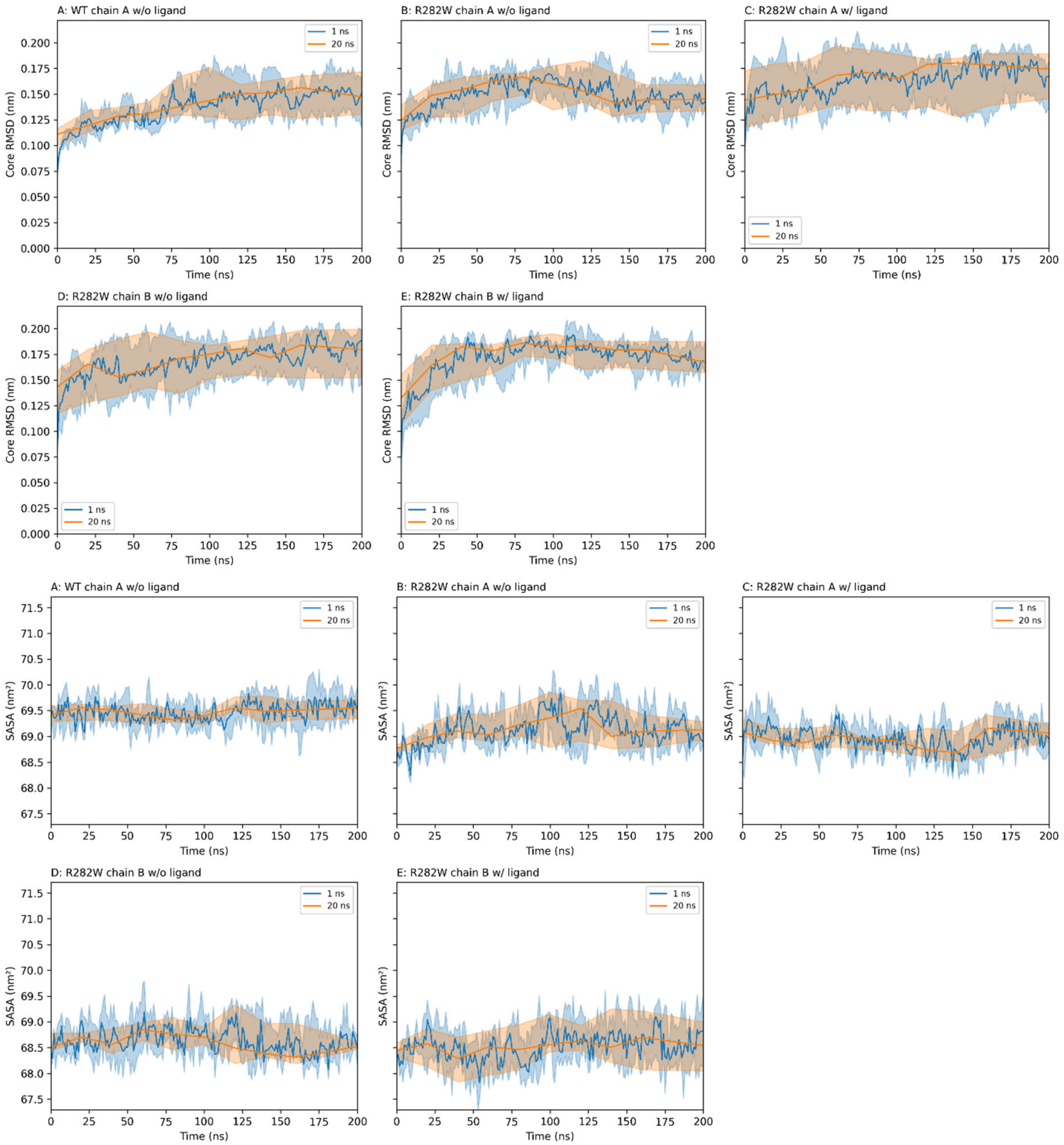

Fig. S4. Solvent accessible surface of the core protein as well as the core RMSD.

To investigate H2 flexibility we tracked the hydrogen bonds between C277-ASP281, P278-R/W282, G279-R283, R280-T284:

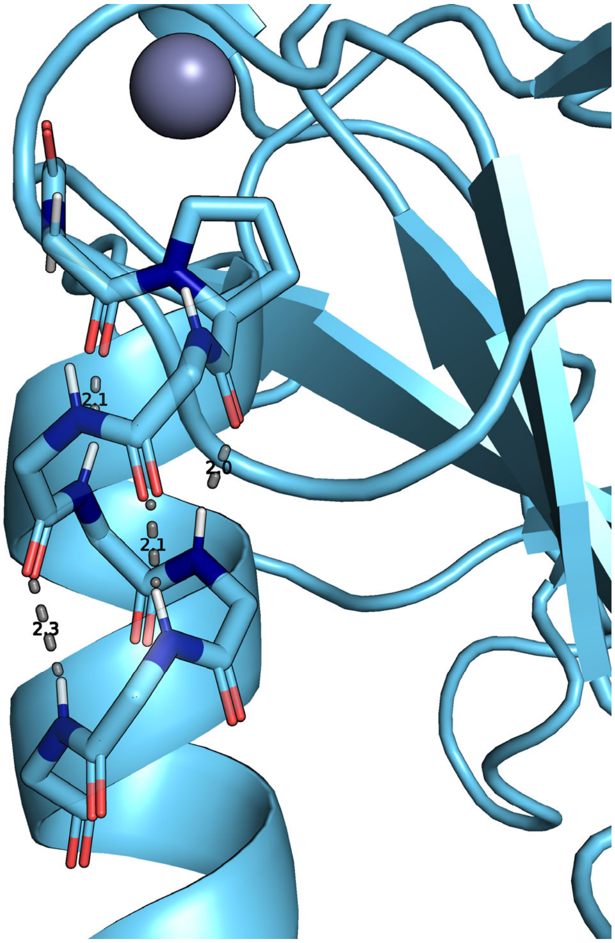

Fig. S5. Representation of the tracked hydrogen bonds.

Chain A and WT showed virtually consistently 4 hydrogen bonds throughout the simulations.

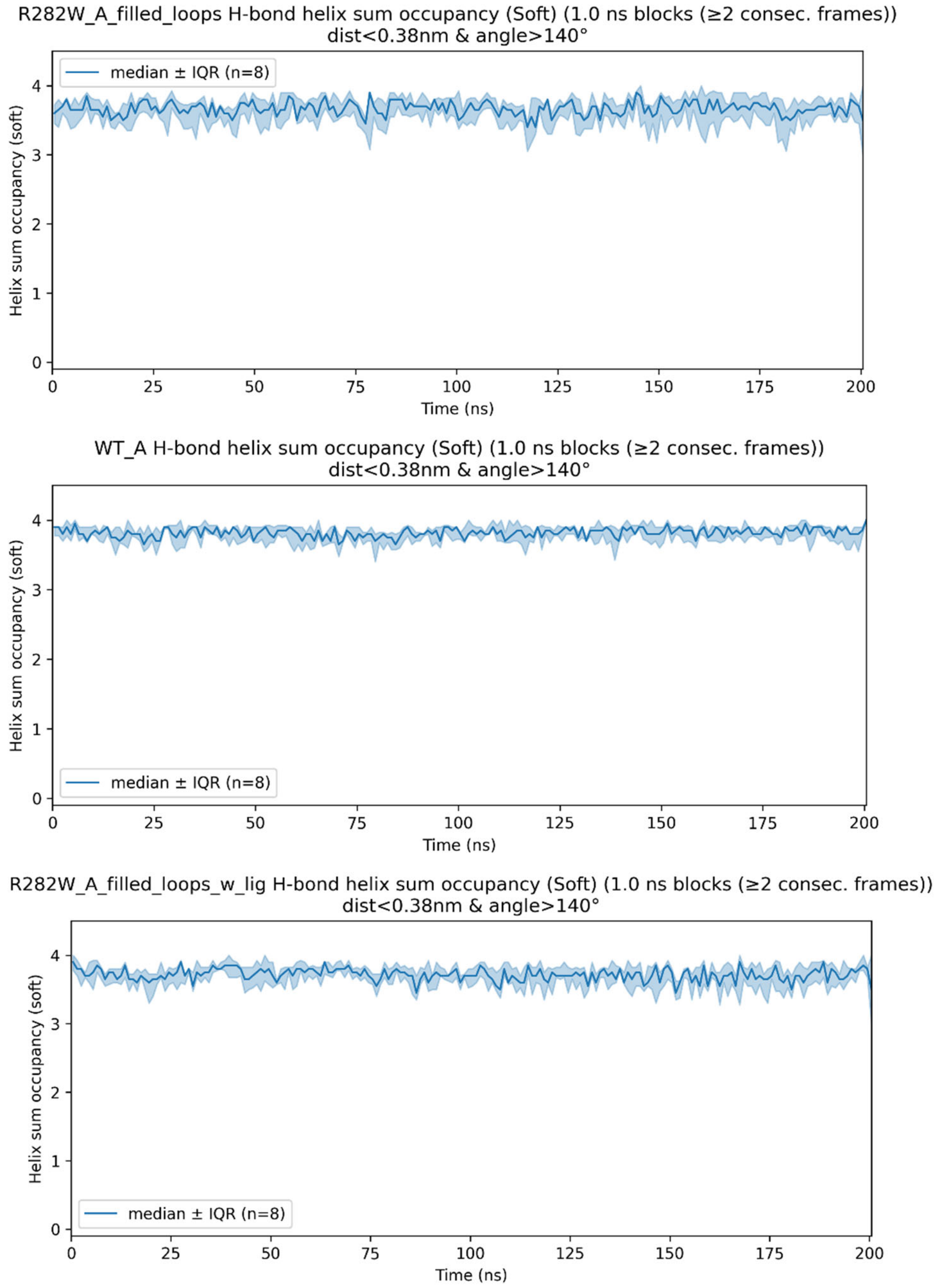

Fig. S6. Amount of hydrogen bonds observed throughout the MD simulation.

Chain B with Ligand shows that the helix remains unwound.

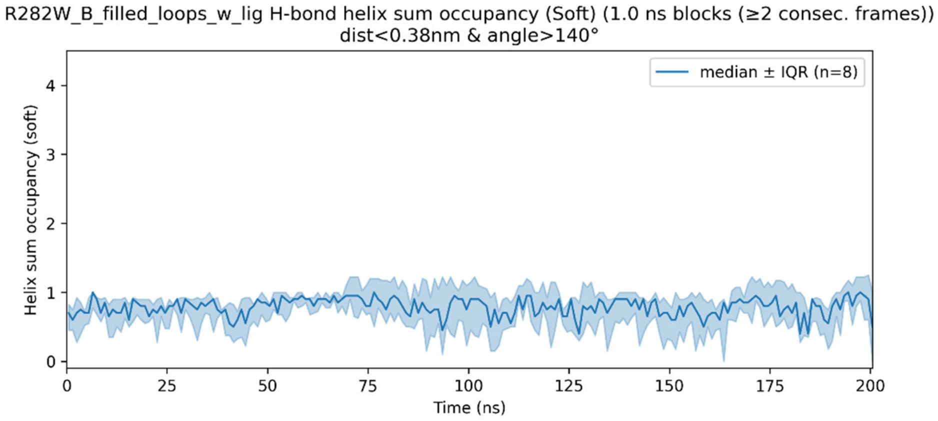

Fig. S7. Amount of hydrogen bonds observed throughout the MD simulation.

For chain B without ligand the ability to rewind the helix differed with particularly replicate 4 restoring all original hydrogen bonds.

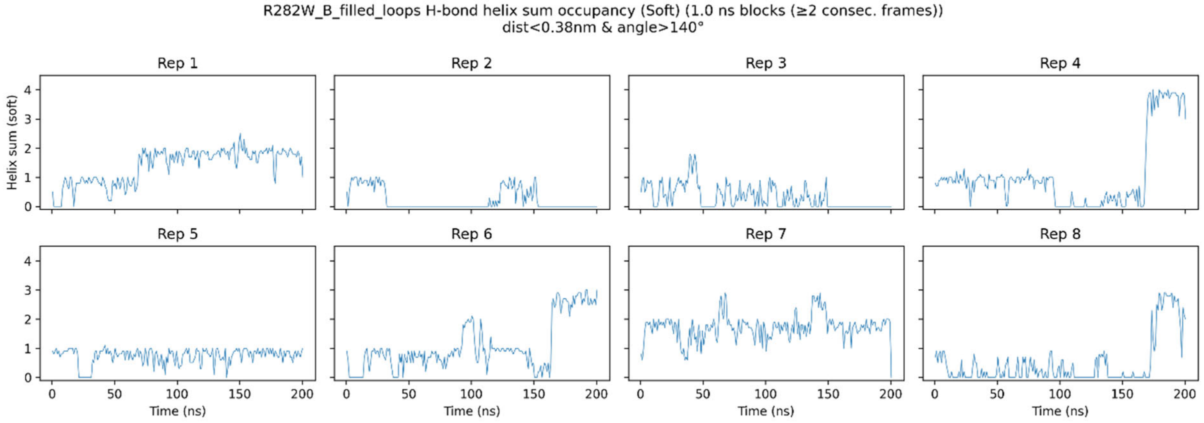

Fig. S8. Display of all 8 replicas of p53-R282W with the ligand removed prior to simulation.

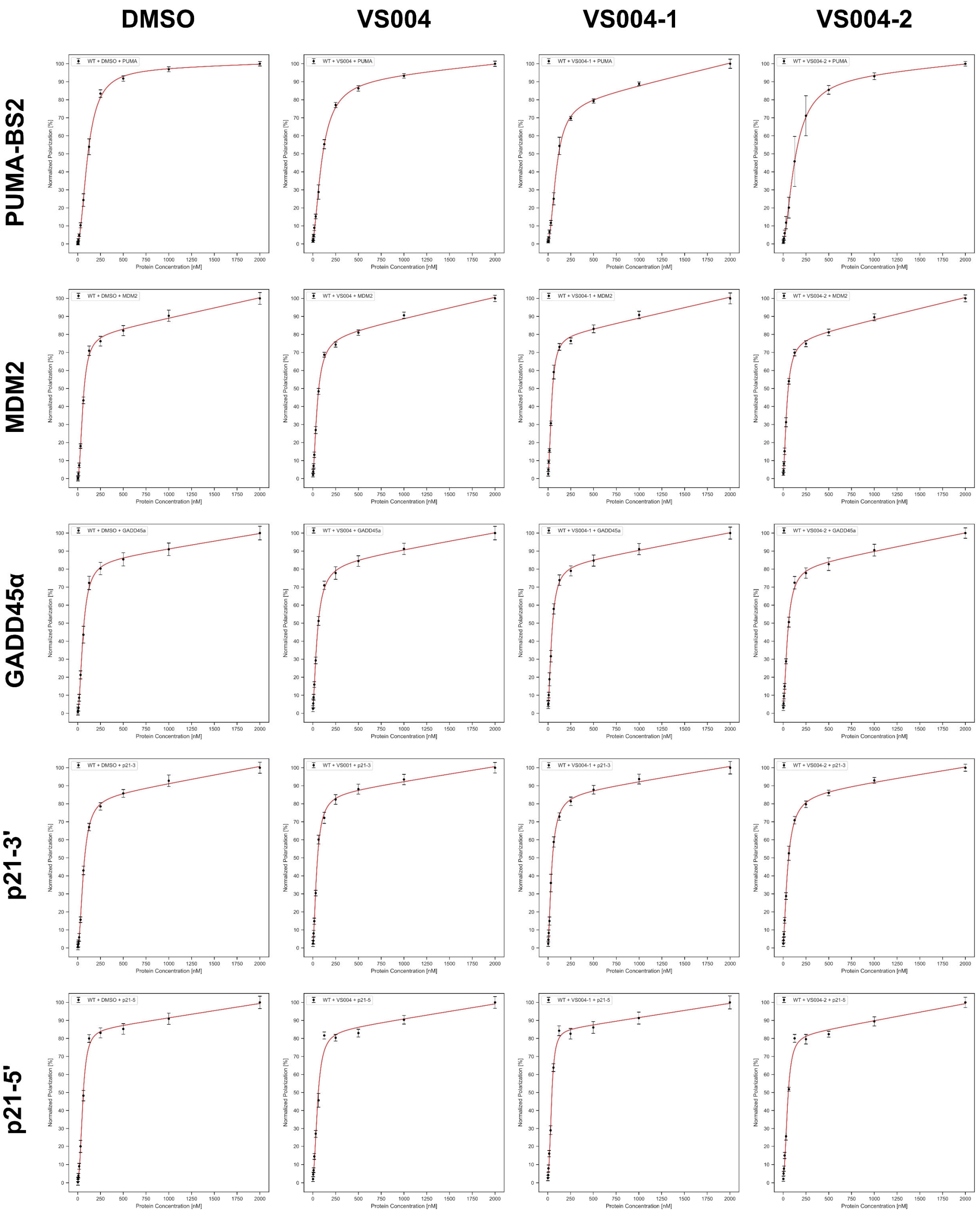

Fig. S9. Plots of the individual fits for K_D fitting of FL-Tp53-WT against different response elements and compounds after 1 h of incubation.

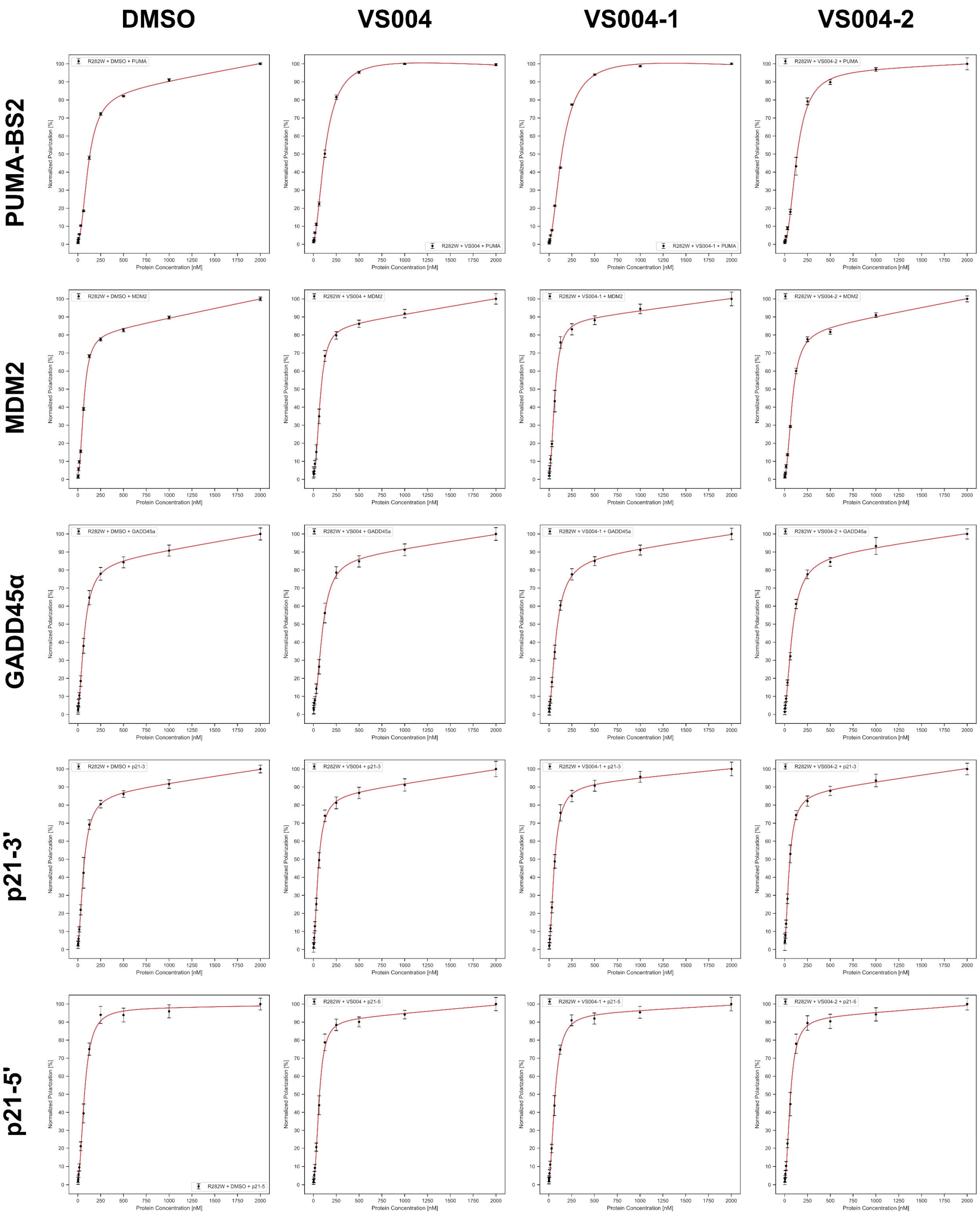

Fig. S10. Plots of the individual fits for K_D fitting of FL-Tp53-R282W against different response elements and compounds after 1 h of incubation.

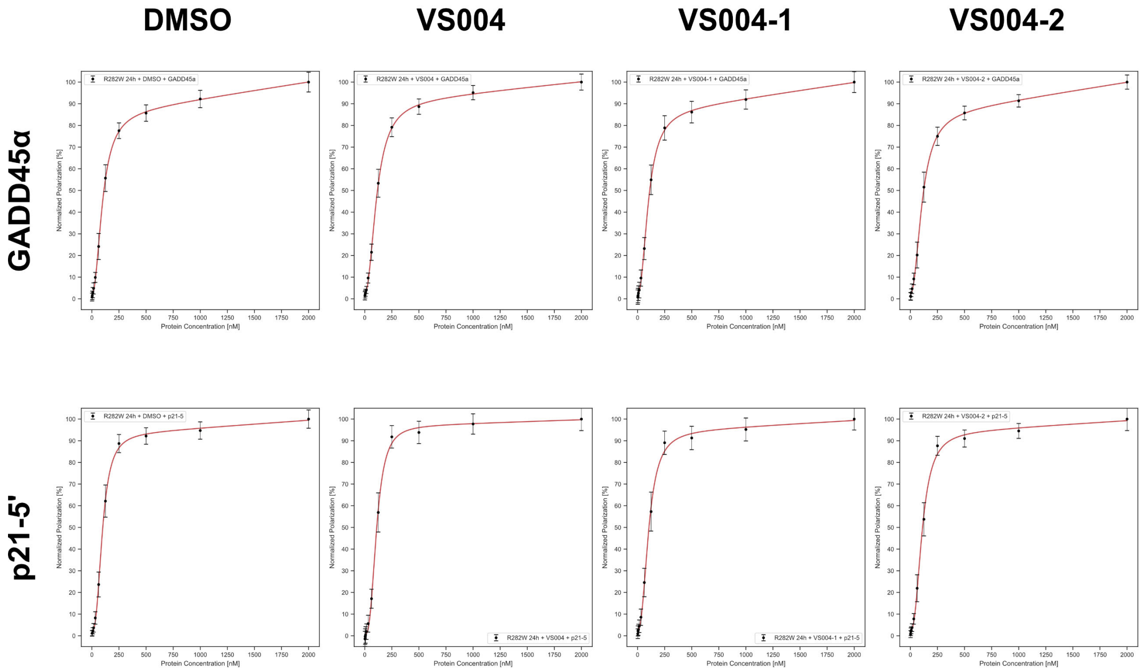

Fig. S11. Plots of the individual fits for K_D fitting of FL-Tp53-R282W against different response elements and compounds after 24 h of incubation.

Table S7. K_D values of the response elements binding to R282W prior to compound or DMSO incubation (24 h). All values are presented in units of nM.

	R282W			
	DMSO	VS004	VS004-1	VS004-2
GADD45α	98 ± 3	110 ± 3	100 ± 3	108 ± 4
P21-5’	96 ± 3	109 ± 4	102 ± 5	109 ± 5
